# Population Genetic Diversity and Phylogenetic Characteristics for High-Altitude Adaptive Kham Tibetan Revealed by DNATyper^TM^ 19 Amplification System

**DOI:** 10.3389/fgene.2018.00630

**Published:** 2018-12-17

**Authors:** Xing Zou, Zheng Wang, Guanglin He, Mengge Wang, Yongdong Su, Jing Liu, Pengyu Chen, Shouyu Wang, Bo Gao, Zhao Li, Yiping Hou

**Affiliations:** ^1^Institute of Forensic Medicine, West China School of Basic Medical Sciences and Forensic Medicine, Sichuan University, Chengdu, China; ^2^Forensic Identification Center, Public Security Bureau of Tibet Autonomous Region, Lhasa, China; ^3^Center of Forensic Expertise, Affiliated Hospital of Zunyi Medical University, Zunyi, China; ^4^School of Forensic Medicine, Zunyi Medical University, Zunyi, China; ^5^Institute of Forensic Science, Yili Public Security Bureau of Xinjiang, Kuytun, China; ^6^Department of Criminal Investigation, Mianyang Public Security Bureau, Mianyang, China

**Keywords:** Tibetan, genetic polymorphism, short tandem repeat, population relationship, forensic genetics

## Abstract

Tibetans residing in the high-altitude inhospitable environment have undergone significant natural selection of their genetic architecture. Recently, highly mutational autosomal short tandem repeats were widely used not only in the anthropology and population genetics to investigate the genetic structure and relationships, but also in the medical genetics to explore the pathogenesis of multiple genetic diseases and in the forensic science to identify individual and parentage relatedness. However, genetic variants and forensic efficiency of DNATyper^TM^ 19 amplification system and genetic background of Kham Tibetan remain uncharacterized. Thus, we genotyped 19 forensic genetic markers in 11,402 Kham Tibetans to gain insight into the genetic diversity of Chinese high-altitude adaptive population. Highly discriminating and polymorphic forensic measures were observed, which indicated that this new-developed DNATyper 19 PCR amplification is suitable for routine forensic identification purposes and Chinese national DNA database establishment. Pairwise genetic distances among the comprehensive population comparisons suggested that this high-altitude adaptive Kham Tibetan has genetically closer relationships with lowlanders of Tibeto-Burman-speaking populations (Chengdu Tibetan, Liangshan Tibetan, and Liangshan Yi). Genetic substructure analyses via phylogenetic reconstruction, principal component analysis, and multidimensional scaling analysis in both nationwide and worldwide contexts suggested that the genetic proximity exists along the linguistic, ethnic, and continental geographical boundary. Further studies with whole-genome sequencing of modern or archaic Kham Tibetans would be useful in reconstructing the Tibetan population history.

## Introduction

Short tandem repeats (STRs), also referred to as microsatellites, are mainly scattered in the non-coding regions of the whole human genome (Gymrek et al., 2017; Willems et al., 2017). This most variable genetic marker in the eukaryotic genomes comprises tandem repeat motif of 2–6 base pairs. The *de novo* mutation rate of STRs is larger several orders of magnitude (approximately 10^-3^–10^-4^) when compared with the binary genetic markers (approximately 10^-8^–10^-9^) of single nucleotide polymorphisms (SNPs) and Insertion/Deletion (InDel) (Willems et al., 2014). STR mutations are generally generated through the molecular mechanism of replication slippage and stepwise mutation model, which can add or subtract one repeat unit (such as the motif of TATC in the D13S317 locus). With the advent of Polymerase Chain Reaction (PCR) in the late 1980s and subsequently tremendous progresses of capillary electrophoresis (CE) and whole-genome sequencing, STRs are broadly used in the disease pathogenesis, genetic diversity, population differentiation, and forensic identification (Willems et al., 2014; Gymrek, 2017). Human population genetic scientists hold the opinions that a large number of factors, such as inbreeding and geographical isolation, migration, gene flow, genetic admixture and population fragmentation, contribute to the genetic diversity of the human genome (Kayser and de Knijff, 2011; Sun et al., 2012). Tishkoff et al. (2009) used 848 microsatellites in over 2,500 individuals to characterize the genetic diversity and dissect the population structure across linguistically, geographically and ethnically diverse African populations, as well as reconstruct the complex human evolutionary history.

In forensic science, multiplex STRs genotyping by the fluorescent labeled PCR amplification combined with the CE approach is recognized as the current gold standard in the personal identification, kinship testing and missing person’s identification (Kayser and de Knijff, 2011). Since second generation multiplex (SGM) including six STRs was used in the establishment of the National DNA Database by the Forensic Science Service (FSS) in England in 1996 (Werrett, 1997), a variety of commercial kits containing 15–25 loci selected from the combined DNA index system (CODIS), expanded CODIS, UK core loci (UCL), German core loci (GCL) and Australia national DNA database (NCIDD), International Criminal Police Organization (INTERPOL) standard set of loci (ISSL) and extended European standard set (ESS-extended) were subsequently developed, validated and applied in the forensic cases (Gill et al., 2006; Hares, 2012). GlobalFiler Express PCR Amplification Kit and Huaxia Platinum System (Thermo Fisher Scientific) are typical systems to increase discrimination power, improve international compatibility and reduce the likelihood of adventitious matches (Wang et al., 2015; He et al., 2018b,e). More recently, a new PCR amplification system, DNATyper^TM^ 19 kit, was developed and validated by the Institute of Forensic Science in the Ministry of Public Security (Beijing, China), which can co-amplify 18 autosomal STRs and one sex-determination marker of Amelogenin focused on Chinese populations.

The Tibetan Plateau is generally considered to have been covered by the ice sheet during the last glacial maximum. Until recently, there has been no consensus view about when colonization began, how Tibetans got there and how they occupied and adapted this cold, arid, hypobaric, and hypoxic environment. Archeological evidence from the Heimahe, Jiangxigou sites suggested that the gradual expansion of foragers’ occupation of Tibet began from 40–25 thousand years ago (kya) (Madsen et al., 2006). Abundant evidence from genetic perspectives documented and reconstructed the Tibetan population history and high-altitude adaptation evolutionary history (Zhao et al., 2009; Qi et al., 2013; Huerta-Sanchez et al., 2014). Zhao et al. (2009) suggested that the matrilineal genetic relics and genetic continuity exist between the Late Paleolithic Tibet inhabitants and modern Tibetans. Genetic analyses of simultaneous testing paternal Y chromosome, maternal mitochondrial DNA and autosomal variations documented the upper Paleolithic occupation and at least one Neolithic expansion (Qi et al., 2013). Additionally, many whole-genome genetic studies have identified that the genetic basis of variations in EPAS1 and EGLN seems to be involved in high altitude adaptation of Tibetans and the corresponding adaptive haplotypes (AGGAA) in the EPAS1 gene are obtained by introgression from Denisovan archaic hominin (Huerta-Sanchez et al., 2014). There are also too many other genetic, linguistic and archeological studies which isolated or combined to reconstruct the complex genetic origin, admixture, divergence with the surrounding populations (Lu et al., 2016; Hu et al., 2017; Zhang et al., 2017; He et al., 2018a,c,f). However, existing genetic data are not sufficient to explore the genetic variations and features of the forensic related markers of Tibetans with different origins and cultural background (Ü-Tsang, Kham and Ando Tibetans).

Thus, we conducted and reported the first large-scale autosomal STRs study in this unique high-altitude adaptive Tibetan population based on a new-generation DNATyper^TM^ 19 PCR amplification system and explored the detailed genetic variants, genetic diversity and forensic efficiency of STRs in the Kham Tibetans in this study. Furthermore, we performed two comprehensive population comparisons (nationwide population relationship investigation among 64 groups and worldwide genetic affinity exploration among 53 groups) to dissect the genetic differentiation between the Kham Tibetans and reference populations and simultaneously provide some new insights for patterns of global or local population substructure based on autosomal genetic variability.

## Materials and Methods

### DNA Sample Collections and Ethics Statements

This project and corresponding protocol were considered and approved by the Ethics Committee of the Institute of Forensic Medicine, West China School of Basic Science and Forensic Medicine, Sichuan University (Approval Number: K2015008). Our participants are needed to be the indigenous Tibetans and no intermarriage or long-distance migration at least three generations. Our subjects have signed written informed consent and analyzed anonymously. A total of 11,402 unrelated healthy individuals (4,846 females and 6,556 males) were collected from the east of Tibet Kham Tibetan autonomous region (Chengdu country), Aba and Muli city in Sichuan province. To insure the included the donors which meet the aforesaid requirement, we followed the following criteria: (1) both parents and grandparents being Tibetans; (2) the language used first is Tibeto-Burman language; (3) all participants residing in the same village or owning the same family names are need to check with relative relationships with previous included subjects to avoid included close relatives; (4) in the past three generations, there is no documented ancestors from other ethnic groups. Besides, to avoid the potential included close relatives, we employed a large sample size to dilute the sample collection bias. Blood samples are collected using FTA cards or cotton swab. All datasets generated and analyzed for this study are included in the Supplementary Material.

### DNA Amplification

Nineteen forensic genetic markers labeled with multi-fluorescent dyes (vWA, TPOX, TH01, Penta E, FGA, D8S1179, D7S820, D6S1043, D5S818, D3S1358, D2S1338, D21S11, D19S433, D18S51, D16S539, D13S317, D12S391, CSF1PO and Amelogenin) were amplified simultaneously using the DNATyper^TM^ 19 PCR amplification system on a GeneAmp PCR System 9700 Thermal Cycler (Applied Biosystems, Foster City, CA, United States) on the basis of the manufacture’s instruction. We employed the following PCR amplification conditions: decomposition at 72°C for 20 min and denaturation at 95°C for 11 min, and then amplification for 26 cycles of denaturation for 30 s at 94°C, anneal for 2 min at 59°C and extension for 1 min at 72°C, following a final extension at 60°C for 60 min, and holding at 25°C. PCR products are mixed with the deionized Formamide and Typer500, and then isolated using the capillary electrophoresis on an ABI 3500 XL Genetic Analyzer (Applied Biosystems, Foster City, CA, United States). Electrophoresis results were visualized and checked using the GeneMapper ID-X Software v1.5 (Applied Biosystems, Foster City, CA, United States).

### Statistical Analysis

The exact tests using a Markov chain of linkage disequilibrium and Hardy–Weinberg equilibrium among 18 forensic autosomal genetic markers, as well as estimation of the observed heterozygosity (Ho) and expected heterozygosity (He), were carried out using the Arlequin version 3.5.2.2^1^ (Excoffier and Lischer, 2010). Online tool of the STRAF (STR Analysis for Forensics) (Gouy and Zieger, 2017) was used to calculate the allelic frequencies and statistical parameters of forensic interest, which included the power of exclusion (PE), probability of matching (PM), polymorphism information content (PIC), and power of discrimination (PD). Population genetic differentiation analyses were conducted in two distinctive reference population panels: nationwide panel and worldwide panel. Pairwise Reynolds genetic distances between the Kham Tibetan and reference populations were calculated using the Phylogeny Inference Package (PHYLIP) version 3.6.7^2^ (Cummings, 2004). Principal component analyses (PCA) on the basis of the allelic frequency distribution of the 18 autosomal STRs among 64 nationwide populations and the 16 autosomal STRs among 53 worldwide populations were carried out using a Multivariate Statistical Package (MVSP) for Windows, version 3.1^3^ (Kovach, 2007). Multidimensional scaling (MDS) plots based on the two pairwise Reynolds genetic distance matrixes were conducted using the IBM SPSS^®^ software^4^ (Hansen, 2005). Finally, two phylogenetic relationships were constructed using the neighbor-joining method in the Molecular Evolutionary Genetics Analysis (MEGA) Version 7.0 (Kumar et al., 2016).

### Quality Control

This study was in accordance with the recommendations of scientific standards for studies in forensic genetics proposed and advocated by the International Society for Forensic Genetics (ISFG) (Schneider, 2007). The experiment was conducted in an ISO 17025 accredited laboratory, which simultaneously passed and accredited by the China National Accreditation Service for Conformity Assessment (CNAS). Laboratory internal standard and manufacturer’s instruction were strictly followed to minimize errors. Negative control (H_2_O) and positive control (9947A) were genotyped along with each batch of samples.

## Results

### Hardy–Weinberg Equilibrium and Linkage Disequilibrium

**FIGURE 1:**
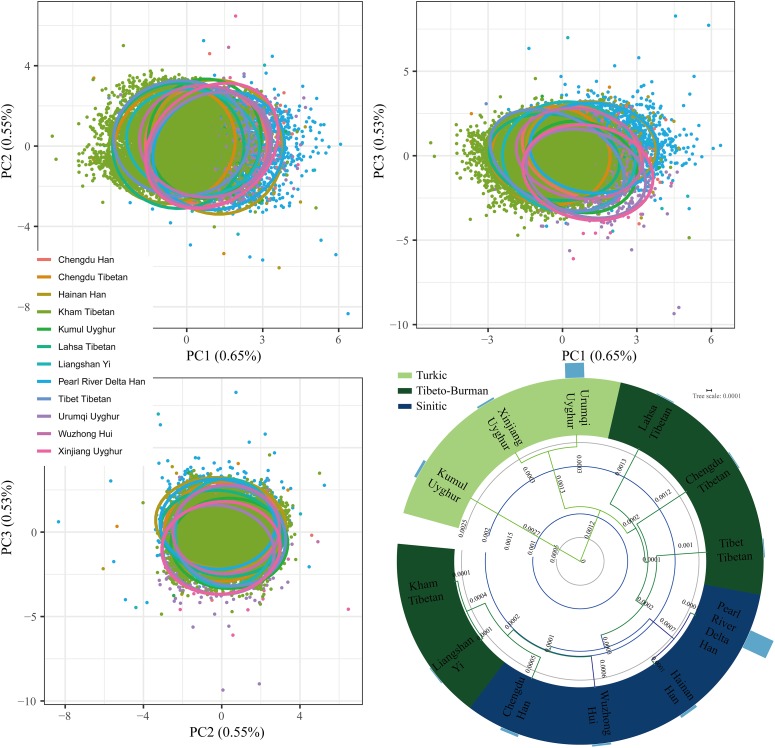
Genetic homogeneity and heterogeneity between Kham Tibetan and other 11 neighboring Chinese populations revealed by principle component analysis and phylogenetic tree. Bar graph (D) denotes the Fst values between Kham Tibetan and corresponding reference populations.

A total of 11,402 Kham Tibetan subjects were successfully genotyped using the DNATyper^TM^ 19 amplification system (Supplementary Table S1). As shown in Table 1, We observed no significant deviation from the Hardy–Weinberg equilibrium (HWE) for the 18 autosomal STRs in Chinese Kham Tibetan after applying the Bonferroni correction for multiple tests (*p* < 0.05/18 = 0.0028). Simultaneously, pairwise Linkage Disequilibrium (LD) among 153 locus pairs was conducted, and we identified 34 pairs existing linkage or associated inheritance in the Kham Tibetan (Supplementary Table S2). To authorize whether population stratifications exit in this Tibetan group. we first test the genetic heterogeneity or homogeneity of the Kham Tibetan via principal component analysis (PCA). As shown in Supplementary Figure S1, 2.23% genetic variations extracted from Kham Tibetan demonstrated that Kham Tibetan is a homogeneous population. To further validate the genetic homogeneity and initially explore genetic similarities with neighboring populations, we conducted a PCA, Fst genetic distance calculation and phylogenetic relationship reconstruction on the basis of raw genotype data of 18 autosomal STRs from 18,499 individuals from 12 populations. As shown in Supplementary Table S3 and Figure 1, a total of 1.73% genetic variations can be extracted by the first three PCs. We identified light population stratifications among geographically and genetically different populations due to most individual plots are overlapped in the PCA analyses. But we can also observe genetic affinity among populations belongs to the same language family (Sinitic, Tibeto-Burman, and Turkic). Generally, population comparisons between the meta-Tibetan and 11 previously investigated populations revealed that Kham Tibetan keep a close genetic relationship with other four Tibeto-Burman-speaking populations (Fst = 0.0001, Supplementary Table S3). Thus, we can establish one database of allele frequency distributions of Tibetan population for forensic routine applications.

**Table 1:** Forensic statistical parameters of 18 forensic autosomal genetic markers in 11,402 unrelated Kham Tibetans residing Tibet Tibetan autonomous region.

Locus	CSF1PO	D12S391	D13S317	D16S539	D18S51	D19S433	D21S11	D2S1338	D3S1358	D5S818	D6S1043	D7S820	D8S1179	FGA	PentaE	TH01	TPOX	vWA
Nall	10	16	9	9	19	16	19	13	9	9	20	11	10	23	21	7	8	9
PIC	0.6824	0.8004	0.7908	0.7355	0.8249	0.8052	0.8222	0.8369	0.6570	0.7071	0.8566	0.7560	0.8059	0.8443	0.9122	0.5751	0.5301	0.7660
PM	0.1188	0.0536	0.0589	0.0878	0.0421	0.0514	0.0439	0.0380	0.1358	0.1031	0.0301	0.0766	0.0518	0.0344	0.0125	0.1884	0.2267	0.0720
PD	0.8812	0.9464	0.9411	0.9122	0.9579	0.9486	0.9561	0.9620	0.8642	0.8969	0.9699	0.9234	0.9482	0.9656	0.9875	0.8116	0.7733	0.9280
PE	0.4661	0.6232	0.6137	0.5322	0.6635	0.6443	0.6714	0.6947	0.4323	0.4932	0.7214	0.5600	0.6483	0.6904	0.8064	0.3095	0.2677	0.5797
TPI	1.8104	2.6725	2.6020	2.1091	3.0116	2.8406	3.0866	3.3320	1.6812	1.9247	3.6592	2.2587	2.8749	3.2840	5.2836	1.2992	1.1909	2.3754
Ho	0.7238	0.8129	0.8078	0.7629	0.8340	0.8240	0.8380	0.8499	0.7026	0.7402	0.8634	0.7786	0.8261	0.8477	0.9054	0.6152	0.5802	0.7895
He	0.7258	0.8227	0.8166	0.7710	0.8417	0.8266	0.8401	0.8533	0.7096	0.7467	0.8698	0.7876	0.8285	0.8593	0.9181	0.6258	0.5847	0.7969
p	0.1753	0.0200	0.0029	0.2376	0.0031	0.0565	0.0043	0.0981	0.3540	0.8699	0.0470	0.0073	0.0631	0.0648	0.0087	0.0332	0.8125	0.4315

*PM, random matching probability; PD, power of discrimination; PIC, polymorphism information content; PE, power of exclusion; TPI, typical paternity index; Ho, observed heterozygosity; He, expected heterozygosity; p, p-value in the Hardy–Weinberg.*

### Genetic Diversities and Forensic Efficiency Parameters

To explore more precise Tibetan-specific allele frequencies for likelihood estimation in the forensic parentage testing and comprehensively evaluate forensic efficiency of the DNATyper^TM^ 19 amplification system in the forensic personal identification, we calculated the allele frequencies of 18 autosomal STRs and corresponding forensic efficiency parameters in this Kham Tibetan population. A total of 238 alleles with corresponding allelic frequencies spanning from 0.00004 to 0.58209 were observed (Supplementary Table S4). FGA was the locus with the most alleles (23) and TH01 had the least gene locus with 7 alleles. The expected heterozygosity, also named as genetic diversity, varied from 0.5847 at locus of TPOX to 0.9181 at locus of Penta E [Average ± Standard (SD): 0.7903 ± 0.0.0852]. Ho values spanned from 0.5802 (TPOX) to 0.9054 (Penta E) (Average ± SD: 0.7835 ± 0.0848). PIC varied from 0.5301 (TPOX) to 0.9122 (Penta E) (Average ± SD: 0.7616 ± 0.0992) and PM varied from 0.0125 (Penta E) to 0.2267 (TPOX) (Average ± SD: 0.0792 ± 0.0568). PD spanned from 0.7733 (TPOX) to 0.9875 (Penta E) (Average ± SD: 0.9208 ± 0.0568) and PE varied from 0.2677 (TPOX) to 0.8064 (Penta E) (Average ± SD: 0.5788 ± 0.1413). TPI spanned from 1.1909 (TPOX) to 5.2836 (Penta E) (Average ± SD: 2.6276 ± 0.9661).

### Population Genetic Diversity Analysis Revealed by Pairwise Reynolds Genetic Distance

**FIGURE 2:**
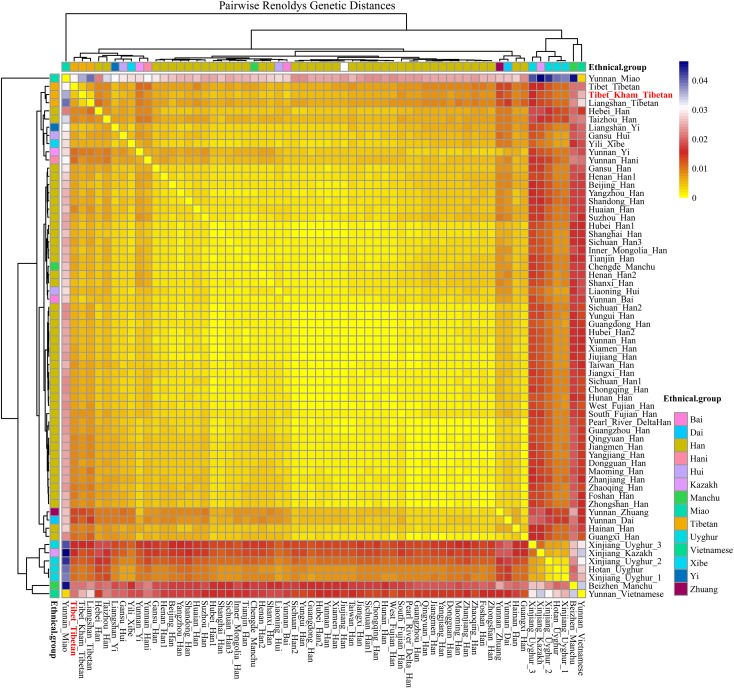
The heat map of pairwise Reynolds genetic distance values for Chinese Kham Tibetan population and the 63 nationwide reference populations with the color scale ranging from yellow, firebrick, white, and navy.

**FIGURE 3:**
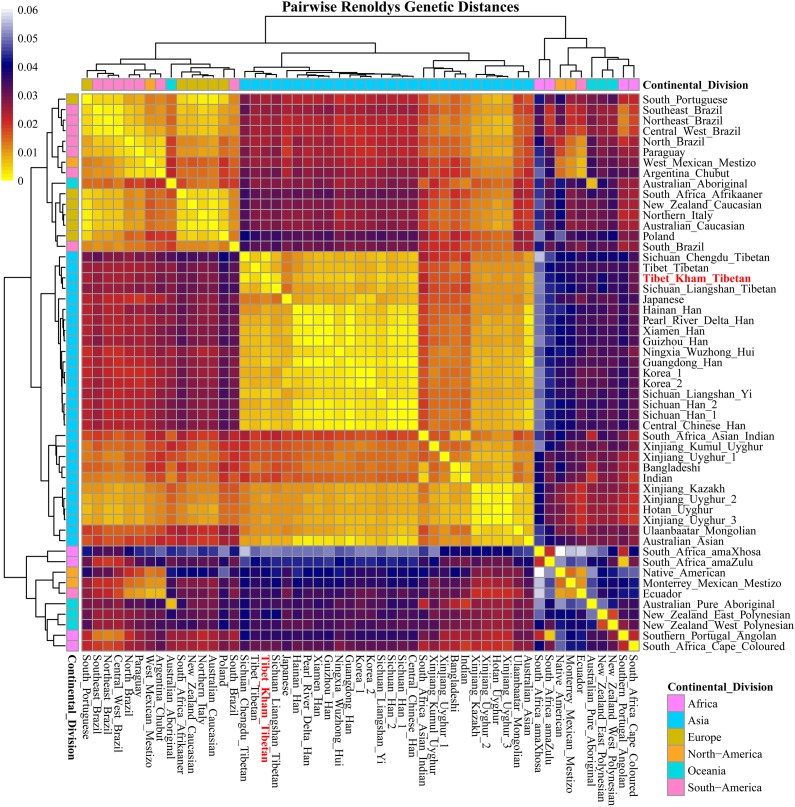
A heat map of pairwise Reynolds genetic distance values of the Tibet Kham Tibetan group and the 52 worldwide comparison populations with the color scale ranging from yellow, firebrick, navy, and white.

For population genetic relationship comparison, we first explored the genetic differentiation between the Kham Tibetan and other 63 Chinese nationwide populations on the basis of 18 overlapped STRs (CSF1PO, D12S391, D13S317, D16S539, D18S51, D19S433, D21S11, D2S1338, D3S1358, D5S818, D6S1043, D7S820, D8S1179, FGA, Penta E, TH0, TPOX, and vWA). This reference population panel comprises of 43 geographically diverse Han Chinese populations, four Uyghur populations, two Manchu, Hui, Yi and Tibetan populations and one Kazakh, Bai, Vietnamese, Miao, Zhuang, Hani and Xibe (Supplementary Figure S2 and Supplementary Table S5). The pairwise Reynolds genetic distances among 64 populations were calculated and presented in Supplementary Table S6. The smallest genetic distance was identified between Jiangxi Jiujiang Han and Yunnan Han (0.0001), followed by Pearl River Delta Han and Guangdong Guangzhou Han (0.0002). The largest genetic one was observed between Xinjiang Kazakh and Yunnan Miao (0.0465). Kham Tibetan had genetically closer relationships with Liangshan Tibetan (0.0035) and Yi (0.0040) and Yunnan Bai (0.0036). A heat map of this genetic matrix showed that the Yunnan Miao and Vietnamese, three Xinjiang Uyghur populations, Kazakh, Benzheng Manchu and Kham Tibetan had overall higher genetic differences compared with the others (Figure 2). To get a worldwide view of genetic similarities and differences of the Kham Tibetan, we made the other comprehensive population comparison which focused on the Kham Tibetan and 52 worldwide reference populations on the basis of 16 overlapped STRs (CSF1PO, D12S391, D13S317, D16S539, D18S51, D19S433, D21S11, D2S1338, D3S1358, D5S818, D7S820, D8S1179, FGA, TH01, TPOX, and vWA). The detailed language family and geographical origins are listed in Supplementary Figure S3 and Supplementary Table S7. Kham Tibetan had a genetic affinity with the Chengdu Tibetan (0.0023) and Liangshan Tibetan (0.0035), followed by Liangshan Yi (0.0039), and owned significant genetic differences with African AmaXhosa (0.0510), AmaZulu (0.0428), and Native American (0.0402). Apparent genetic affinity within-continent populations, such as East Asians, can be detected in the heat map (Supplementary Table S8 and Figure 3).

### Principal Components Analyses Among 64 Nationwide and 53 Worldwide Populations

**FIGURE 4:**
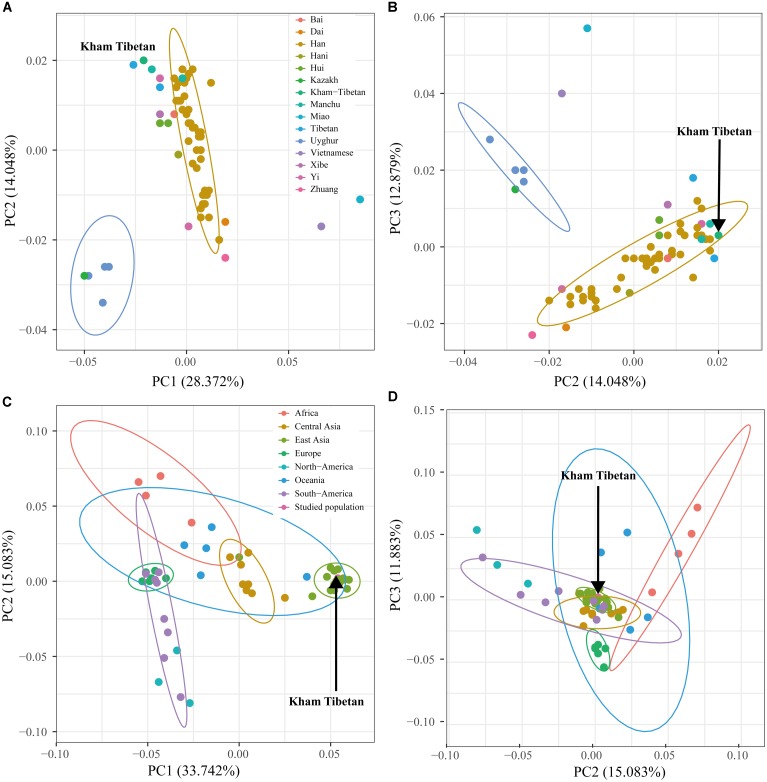
Principal component analyses (PCA) showed the genetic relationships between the Kham Tibetan and reference populations. **(A,B)** PCA was constructed on the basis of the first three components extracted from the allelic frequency distribution of 18 autosomal STRs among 64 Chinese nationwide populations. **(C,D)** PCA was established on the top three components from genetic polymorphisms of 16 autosomal STRs among 53 worldwide populations. Population name abbreviations are in accordance with Supplementary Table S8.

Principal component analyses based on the genetic data has been widely used in correcting for population stratification to avoid false negative or positive results in the genome wide association studies, making qualified ancestry inferences in the human history reconstruction and detecting population substructure (Patterson et al., 2006; Pickrell and Pritchard, 2012). We first performed PCA among 64 populations on the basis of the allelic frequency distribution (Figures 4A,B). The top 10 components could extract a total of 73.390% genetic variants (PC1: 28.372%; PC2: 14.048%; PC3: 12.879%; PC4: 8.230%; PC5: 3.892%; PC6: 2.906%; PC7: 2.697%; PC8: 2.357%; PC9: 2.123%; PC10: 1.885%). Figure 4A was constructed on the basis of the first two components. We observed a clear separation between Han Chinese populations and minority ethnicities with the exception of the Chengde Manchu and Yunnan Bai. PC1 separated Yunnan Miao and Vietnamese from the others, and PC2 separated five Turkic-speaking populations from others. Tibet Kham Tibetan could be distinguished by both PC1 and PC2, and located in the left corner of the first quadrant upper. We subsequently explored the population substructure among 53 worldwide populations via the genetic polymorphisms of 16 polymorphic STRs (Figures 4C,D). Around 84.663% genetic variants had been extracted by the first 10 components. The PC1 to PC10 were, respectively, consisted of 33.742, 15.083, 11.883, 6.524, 5.303, 3.119, 2.985, 2.164, 2.023, and 1.837%. Synthetic map based on the combination of PC1 and PC2 was presented in Figure 4C. East Asian populations were distinguished in the PC1 and three African origin populations and seven European populations were separated in the PC2.

### Multidimensional Scaling Analyses

**FIGURE 5:**
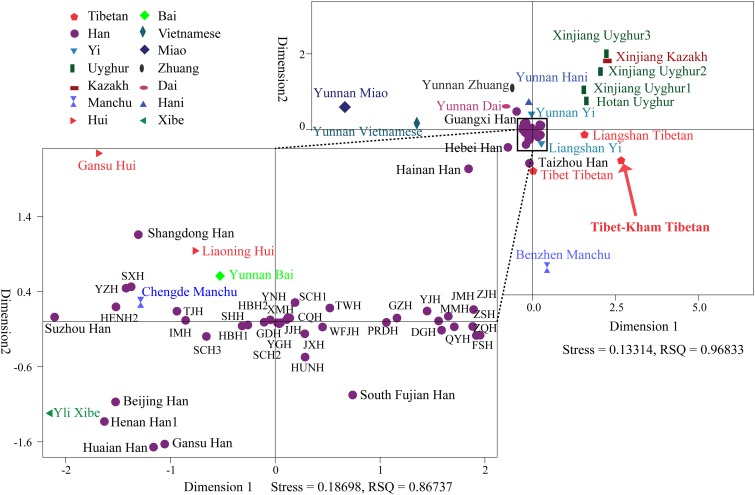
Multidimensional scaling analysis performed based on the pairwise Reynolds values for Kham Tibetan group and 63 reference populations. Population name abbreviations are in accordance with Supplementary Table S6.

To further illustrate and dissect the genetic relationships between the Tibet Kham Tibetan and 63 nationwide groups, as well as 52 worldwide populations, we performed multidimensional scaling analyses using the national-scale and world-scale pairwise genetic distance matrixes. As shown in Figure 5, Kham Tibetan was localized close with Liangshan Tibetan and Tibet Tibetan and located alone in the fourth quadrant of the coordinate axis. Han Chinese populations, except for Guangxi Han, Hebei Han and Taizhou Han, fell close to each other and were generally close to four Chinese minority ethnicities (Gansu Hui, Liaoning Hui, Yunnan Bai, and Yili Xibe). Other Chinese minorities, including five Turkic-speaking populations (Uyghur and Kazakh), five Tibetan-Burman-speaking populations (Tibetan and Yi), Miao, Zhuang, Dai and Vietnamese, formed a loose cluster and distinguished from Han Chinese populations.

**FIGURE 6:**
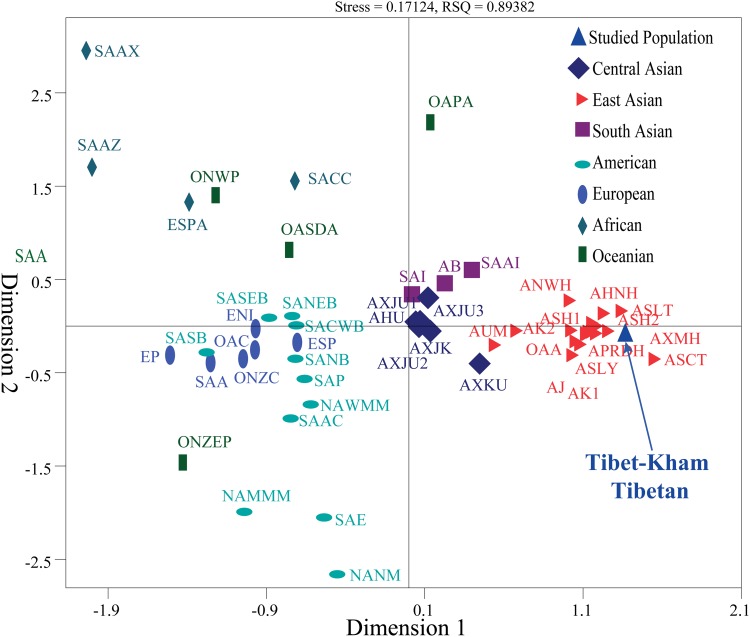
Multidimensional scaling analysis revealed the genetic similarities and differences between the Tibet Kham Tibetan and other 42 reference populations. Population name abbreviations are in accordance with Supplementary Table S8.

We also carried out a new MDS which projected worldwide populations. It is evident that the worldwide population substructures were concordant with continental boundaries (Africa, Europe, South Asia, Central Asia, East Asia, America, and Oceania), which is in accordance with the observed patterns of population genetic relationship in the PCA (Figure 6).

### Phylogenetic Reconstruction Among Two Datasets

**FIGURE 7:**
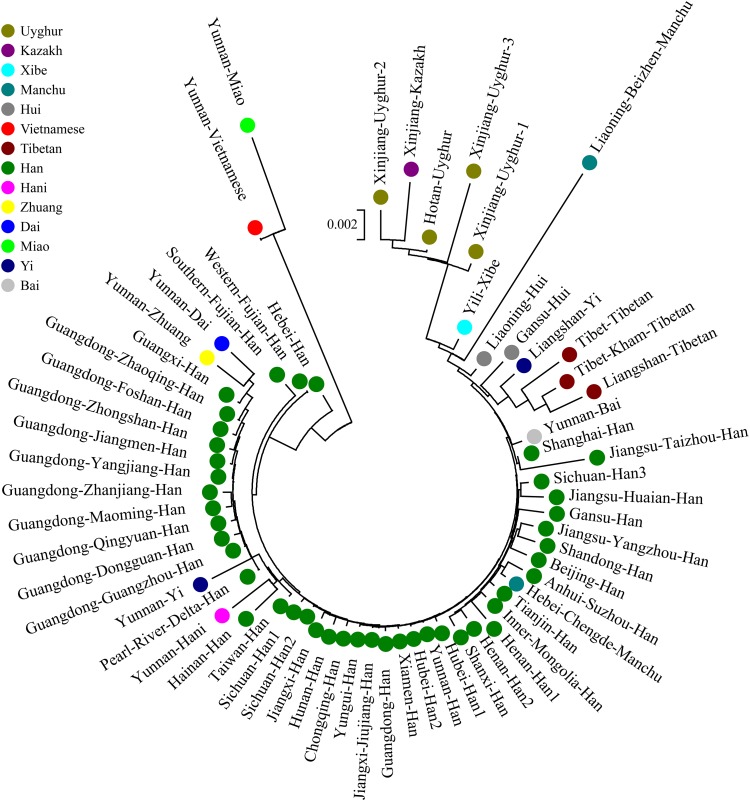
A phylogenetic tree conducted based upon Reynolds distance values of the Kham Tibetan and 63 comparison groups.

**FIGURE 8:**
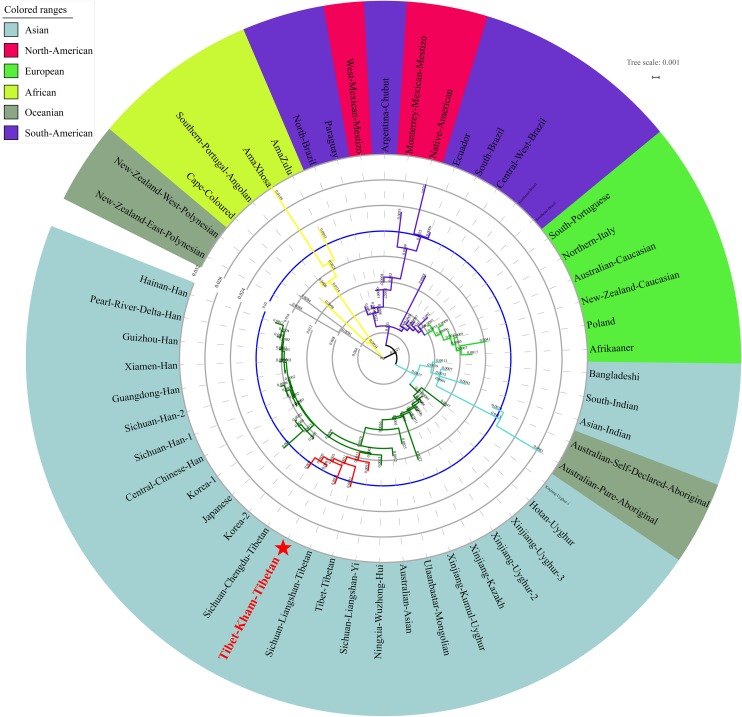
A neighbor-joining tree showed the phylogenetic relationship between the Kham Tibetan and 52 worldwide reference populations.

We finally carried out phylogenetic relationship reconstructions on the basis of the neighbor-joining (N-J) algorithm. A N-J tree based on the pairwise Reynolds’ genetic distance among 64 Chinese populations (Supplementary Table S6 and Figure 7) suggested that the Tibet Kham Tibetan was genetically closer to the surrounding Tibeto-Burman-speaking populations. Kham Tibetan was first grouped with Liangshan Tibetan, and then subsequently grouped with Tibet Tibetan and Liangshan Yi. Tibeto-Burman-speaking genetic affinity cluster was first pooled with the Chinese minority cluster, which consisted of six Altaic-speaking populations, Benzhen Manchu, and finally pooled the Han Chinese populations’ cluster which was mixed with several ethnic minorities (Chengde Manchu, Yunnan Bai, Hani, Yi, Bai, and Zhuang). Six populations were served as the outliers in this N-J tree (two Sichuan Han populations, Hebei Han, Yunnan Vietnamese and Miao). A continuity phylogenetic relationship reconstruction was performed between the Kham Tibetan and a large set of contemporary worldwide populations. Figure 8 showed that a genealogical link was located mainly in close linguistic, ethnical and geographical proximity. Linguistic proximity could be evidently observed in Asian populations, which included Sinitic-, Tibeto-Burman-population cluster in the East Asia, Altaic-speaking populations in the Central Asia, Indo-European-speaking groups in the Europe and so on. Populations from one continent or language family are genetically closer to each other than other geographically or linguistically diverse populations. Genetic similarities from continentally different populations were observed between south Asian Indian and South African Indian, South Portugal Angolan and African Cape-Colored, African Afrikaner and Polish, New Zealand and Australian Caucasian and European Caucasian. All of these populations with ethnic proximity had recent large-scale population colonization, migration and genetic admixture.

## Discussion

### Genetic Polymorphisms and Forensic Characteristics of Kham Tibetan

The characterization and identification of genetic diversity of forensic genetic markers across ethnically diverse populations are important before employing one kind of markers or one amplification system in the forensic cases. Knowledge of the frequency and distribution of forensic markers (SNPs, STRs, insertion/deletion, multi-InDel, microhaplotype and so on) should be accurately obtained and understood to evaluate the forensic efficiency and paternity probability. Ho, He, and PIC values observed in this study indicated that the 18 autosomal STRs are high diversity and polymorphic in the Tibet Kham Tibetan. The overall forensic efficiency values of the combined power of discrimination (CPD) and the combined probability of exclusion (CPE) are 0.99999999999999999999974 and 0.999999931, respectively. This new PCR amplification system is more polymorphic and informative compared with the forensic effectiveness of 21 non-Combined DNA Index System (CODIS) autosomal STRs included in the AGCU 21+1 system, which CPD and CPE are, respectively, 0.9999999999999999999 and 0. 999997 in the Liangshan Tibetan and 0.9999999999999999993 and 0.999999 in the Liangshan Yi (He et al., 2018f). Simultaneously, the discrimination and exclusion powers of this new-developed system in the Kham Tibetan are better than the previously wide-used AmpFlSTR^®^ Sinofiler^TM^ kit, in which CPD and CPE values in 1,220 Tibetans are 0.9999999999999999997 and 0.9999996, respectively (He et al., 2018c). Moreover, the forensic efficiency is also better than 19 X-chromosomal STRs included in the AGCU X19 kit in the Tibetan population (He et al., 2018a). Thus, next-generation autosomal STRs amplification system of DNATyper^TM^ 19 is suitable for the routine forensic applications: individual identification, parentage testing, the national database establishment, missing person identification and so on.

### Genetic Relationships Between Tibetan and Nationwide or Worldwide Reference Populations

Microsatellites with the features of easy typing and availability of large numbers have been widely used to study the genetic diversity, relationship among different human populations. A previous simulation genetic study conducted by Nei and Takezaki (1996) suggested that a more reliable phylogenetic relationship within closely related populations than between distantly related groups could be revealed by microsatellite loci. Thus, we carried out the PCA, MDS and N-J phylogenetic relationship reconstruction on the basis of genetic variations of two datasets (one dataset comprises of 18 autosomal STRs in 64 nationwide populations, and the other one consists of 16 autosomal STRs in 53 worldwide populations) to obtain an overview of genetic relationships, population substructure of Tibetans and adjacent populations. Pairwise Reynolds genetic distances indicated an affinity between the Kham Tibetan and other Tibeto-Burman-speaking populations, including Liangshan Tibetan and Yi, Chengdu Tibetan and Tibet Tibetan, suggesting their similar origin and the natural selection process. Comparisons of nationwide to worldwide genetic variation distribution also showed the significant genetic distinctions between Han Chinese populations and other East Asians and other continental residing groups. Our findings further confirmed the patterns of diversity and substructures revealed by ancestry-informative markers (Wang Z. et al., 2018), and previous population genetic findings (Qi et al., 2013; Huerta-Sanchez et al., 2014; Lu et al., 2016; Wang L.X. et al., 2018). Zhang et al. (2017) suggested Tibetan and Han Chinese populations are diverged at 6.2–16 kya and subsequently diverged with adjacent Sherpa at 3.2–11.3 kya. Recent genetic studies indicated that at least four modern ancestry sources (East Asian, South Asian, Central Asian and Siberian, and western Eurasian and Oceanian) and four archaic ancestry sources [Neanderthal-like, Denisovan-like, ancient-Siberian-like, and even unknown ancestries which is a part of Non-modern human sequences or archaic-like signals in Tibetan gene pool identified by the S^∗^ method (Browning et al., 2018) with the exception of aforementioned three components] exist in the modern Tibetan, as well as revealed at least two Neolithic expansions and one Paleolithic colonization (Qi et al., 2013; Huerta-Sanchez et al., 2014; Lu et al., 2016; Wang L.X. et al., 2018). These complex processes of demographic population history and genetic adaptation shaped the unique population relationship observed in the present study of this high-altitude adaptive Tibetan population.

### Population Substructure in China

Our results showed that Han Chinese populations – long believed to the decedents of Yanhuang Emperors who shared similar cultural artifacts and underwent several southward migrations as well as an admixture with southern indigenous minorities – presented a population stratification (Wen et al., 2004). Significant genetic difference between North-China Han and South-China Han was identified, which is consistent with the earlier research findings via maternal mitochondrial, paternal Y-chromosomal and autosomal genetic materials (Chen et al., 2009; Xu et al., 2009; Nothnagel et al., 2017; Chiang et al., 2018). This North-to-South cline is dependably supported by our heat map, MDS, N-J phylogenetic relationship reconstruction and PCA analyses, as well as illustrated by the low pairwise Reynolds’ genetic distance within South Han Chinese populations and Northern Han Chinese populations and larger genetic distance between them. China is a country which is rich in the genetic, linguistic, geographical, ethnical, and cultural diversity. There are 55 officially recognized minority ethnicities and Han Chinese, which belong to seven language families [Tai-Kadai, Hmong-Mien, Sino-Tibetan (Sinitic branch and Tibeto-Burman branch), Altaic (Tungusic, Turkic and Mongolic), Austroasiatic; Indo-European and Austronesian] consisting of over 290 different recognized languages. Our population genetic comparison analyses simultaneously revealed that most minorities, especially for Altaic-speaking and Tibeto-Burman-speaking populations, possess different genetic ancestry components at varying degrees compared with other references. In our PCA and MDS analysis, we found most of the minorities isolated and scattered compared with the tight close Han Chinese cluster. These findings are congruent with the appearance of unique local climate (High-altitude in Tibet) and intermarriage within the same cultural background and clan beliefs (Turkic-speaking populations in northwestern China). In general, separated ethnic-specific origins (56 ethnicities), enormous geographic separation (the Yangzi and Yellow Rivers as well as the Himalayas), potentially existing ongoing and substantial gene flow among ethnically, geographically and linguistically different populations may serve as the Chinese plausible demographic mechanisms to explain the patterns of genetic variations.

### Worldwide Population Genetic Similarities and Differences via Autosomal STRs

The migration routes and time of the human out of Africa have been subsequently discovered and validated using patterns of genetic variation in the maternally inherited mitochondrial DNA, paternally inherited Y chromosome and autosomal chromosome. Dramatic events accompanied by the changes in cultural interactions and social structure in prehistoric and historic times, such as worldwide Hunter-Gatherer transition, Bantu expansion in Africa, Agriculture spread from Anatolia to Europe and complex Neolithic/Bronze Age migrations from the Pontic-Caspian Steppe in Europe, Mongol Empire expansion in Eurasia and complex migrations in Oceania and America, have shaped the worldwide genomic variations of anatomically modern human (Nielsen et al., 2017). Nowadays, the sharing data with larger sample size and global population coverage in forensic science provided an opportunity to investigate the worldwide population relationship and substructure. Our results from comparative studies across 53 worldwide ethnically diverse human populations have revealed numerous genetic affinity clusters, including the Asian cluster, American cluster, European cluster, African cluster, and Oceanian cluster. Our findings are consistent with the accumulation of population- or region-specific genetic variability under the human adaptation model of “going global by adapting the local” (Fan et al., 2016). We observed obvious genetic affinity among intra-continental populations and genetic differentiation among inter-continental populations. Although geographical structuring of worldwide populations at the continental level can be ideally identified via this simple sequence repeat, no expected genetic relationships between continental populations is observed. In this study, African and Oceanian populations clustered first in the MDS, PCA and N-J tree. Africa has substantial ethnic, cultural and linguistic diversity, which is the origin of anatomically modern humans and the source of the worldwide range modern human expansion (Beltrame et al., 2016). Cape-Colored, AmaXhosa, AmaZulu and Southern Portugal Angolan clustered with New Zealand Polynesians. Polynesians distributing across a triangle of islands in the South Pacific are descendants of mixed Melanesian and East Asian ancestry. Besides, European and American grouped first and European populations kept genetic affinity with each other, including two immigrant Caucasian groups living in Australia and New Zealand. Anatomically modern humans started residing in Europe from 43 kya and underwent different genetic ancestry component admixtures and even population turnover (Damgaard et al., 2018; Mathieson et al., 2018; Olalde et al., 2018). The peopling of the indigenous American lately started approximately 15 kya via the Eurasia and Bering Strait, and then subsequently expanded and widespread settled in the North and South America (Raghavan et al., 2015; Moreno-Mayar et al., 2018). Generally, Africans and Oceanians are both remotely related to Asian, American, and Europeans in the tree, so they clustered together as kind of outliers. Recent population genomic studies on the basis of genetic variants of modern and ancient peoples has also demonstrated that southern Africans are a deep lineage of modern humans (Skoglund et al., 2017) and interbreeding between anatomically modern humans (Europeans, Asians, and Oceanians) and extinct hominins (Neanderthal or Denisovan) occurred (Nielsen et al., 2017; Browning et al., 2018). Beside of these ethnical-specific genetic components contributed our observed patterns, other limitations in the population comparison analyses should be with cautions in understanding population relationships: (1) Mestizos included in our included populations may be influenced the patterns of genetic relationship; (2) the included populations and marker panel density are small and more genetic information of demographically, culturally and linguistically representative is lack; (3) it is well known that high-mutated genetic marker are better used to investigate genetic history in the genetically close populations (intra-continental populations) and have limitations in precisely dissecting genetic structure in geographically isolated for a long time. In Asia, we evidently observed three Asian sub-clusters, which included the Sinitic-speaking, Turkic-speaking, and Altaic-speaking clusters. The patterns of genetic affinity are in accordance with language family boundaries, and are confirmed our previously observed genetic heterogeneity and homogeneity revealed by ancestry-informative single nucleotide polymorphisms (He et al., 2018d; Wang Z. et al., 2018), Y-chromosomal STRs (He et al., 2017a) and X-chromosomal STRs (He et al., 2017b,c, 2018a).

## Conclusion

In summary, we presented the first batch population data of large sample size (11,402) to comprehensively evaluate the genetic diversity and forensic efficiency of DNATyper^TM^ 19 PCR amplification system in the Kham Tibetan population. Ideal forensic measures observed in this study indicated that the 18 forensic autosomal genetic markers are polymorphic, informative and useful in forensic personal identification, parentage testing and national database establishment in Chinese Kham Tibetans. Additionally, we employed a total of 64 Chinese nationwide populations and 53 worldwide populations as two reference panels to explore and clarify the genetic origin, genetic relationships between the Kham Tibetan and reference populations. Our comparative analysis results demonstrated that this high-altitude adaptive Kham Tibetan has genetically closer relationships with low-altitude residing Tibeto-Burman-speaking populations (Chengdu Tibetan, and Liangshan Tibetan and Yi). Finally, genetic substructure analyses in the nationwide and worldwide context suggested that the genetic proximity exists along with linguistic, ethnic, and continental geographical boundary. Additional studies with whole-genome sequencing of modern or archaic Kham Tibetans would help in reconstructing Tibetan population history.

## Ethics Statement

This study was carried out according to the Declaration of Helsinki and the recommendations of “Ethical Committee of Sichuan University, China” with written informed consent from all subjects. Our protocol was approved by the “Ethical Committee of Sichuan University” (Approval Number: K2015008).

## Author Contributions

XZ and GH wrote the manuscript. MW, JL, PC, BG, SW, and ZL collected the samples and extracted DNA. GH, MW, XZ, and JL helped to conduct the statistical analysis. ZW revised the manuscript. YH designed this study. All authors agreed to the submission of the manuscript.

## Conflict of Interest Statement

The authors declare that the research was conducted in the absence of any commercial or financial relationships that could be construed as a potential conflict of interest.

## Footnotes

1 http://cmpg.unibe.ch/software/arlequin35/

2 http://evolution.genetics.washington.edu/phylip.html

3 https://www.kovcomp.co.uk/mvsp/index.html

4 https://www.ibm.com/analytics/spss-statistics-software
