# Supplementary material for: Population Genetic Diversity and Phylogenetic Characteristics for High-Altitude Adaptive Kham Tibetan Revealed by DNATyperTM 19 Amplification System
Source: Front Genet. 2018 Dec 17;9:630. doi: 10.3389/fgene.2018.00630 (PMC6304359; doi:10.3389/fgene.2018.00630)
Supplement: Supplementary file 12 [file Data_Sheet_3.docx]

**
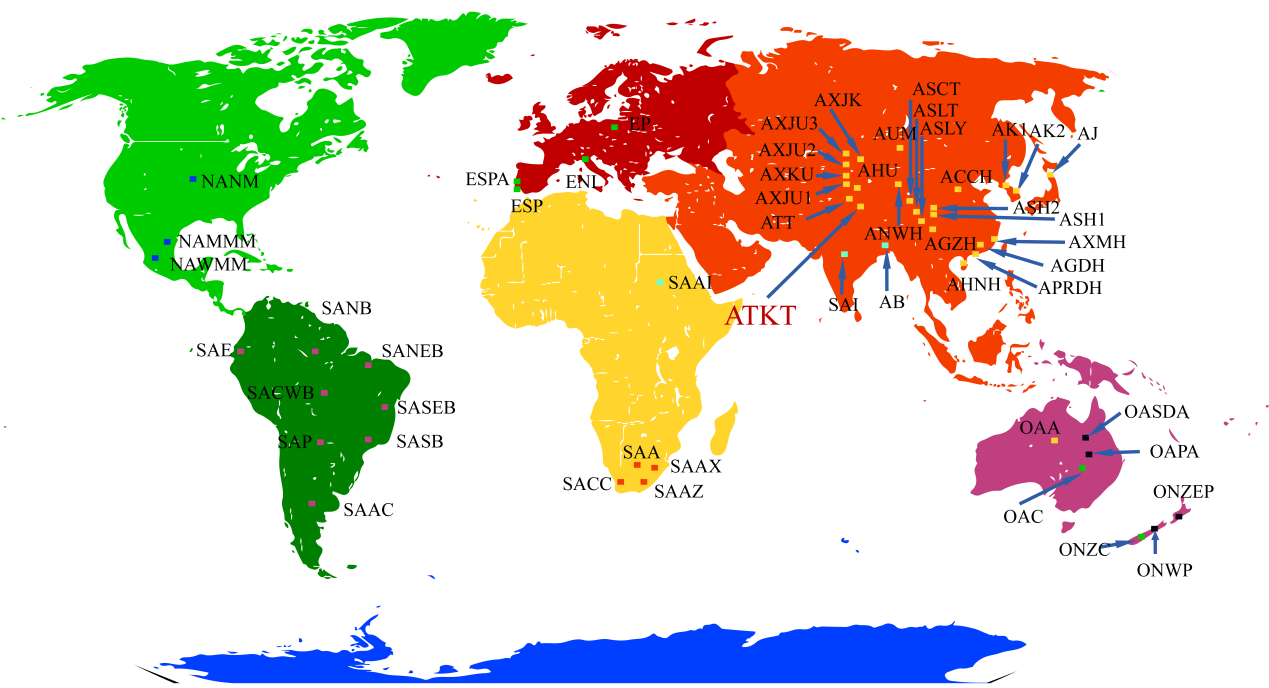
Figure S3**. The geographical positions of 53 included populations in the worldwide population genetic relationship investigation.
